# Comparative Analysis of Volatile Compounds in the Flower Buds of Three *Panax* Species Using Fast Gas Chromatography Electronic Nose, Headspace-Gas Chromatography-Ion Mobility Spectrometry, and Headspace Solid Phase Microextraction-Gas Chromatography-Mass Spectrometry Coupled with Multivariate Statistical Analysis

**DOI:** 10.3390/molecules29030602

**Published:** 2024-01-26

**Authors:** Yang Yue, Jiaxin Yin, Jingyi Xie, Shufang Wu, Hui Ding, Lifeng Han, Songtao Bie, Wen Song, Ying Zhang, Xinbo Song, Heshui Yu, Zheng Li

**Affiliations:** 1 College of Pharmaceutical Engineering of Traditional Chinese Medicine, Tianjin University of Traditional Chinese Medicine, Tianjin 301617, China; 15635410471@163.com (Y.Y.); yiyi526117@163.com (J.Y.); xiejingyi199709@163.com (J.X.); wsf199807@163.com (S.W.); dinghui.hn@163.com (H.D.); hanlifeng_1@sohu.com (L.H.); biesongtao@126.com (S.B.); songxinbo@tjutcm.edu.cn (X.S.); 2 Haihe Laboratory of Modern Chinese Medicine, Tianjin 301617, China; 3 State Key Laboratory of Component-Based Chinese Medicine, Tianjin University of Traditional Chinese Medicine, Tianjin 301617, China; 4 Tianjin HongRenTang Pharmaceutical Co., Ltd., Tianjin 300385, China; hrtjishu@163.com (W.S.); gblzhangying@163.com (Y.Z.)

**Keywords:** *Panax*, flower bud, fast GC e-nose, HS-GC-IMS, HS-SPME-GC-MS, multivariate statistical analysis

## Abstract

The flower buds of three *Panax* species (PGF: *P. ginseng*; PQF: *P. quinquefolius*; PNF: *P. notoginseng*) widely consumed as health tea are easily confused in market circulation. We aimed to develop a green, fast, and easy analysis strategy to distinguish PGF, PQF, and PNF. In this work, fast gas chromatography electronic nose (fast GC e-nose), headspace-gas chromatography-ion mobility spectrometry (HS-GC-IMS), and headspace solid phase microextraction-gas chromatography-mass spectrometry (HS-SPME-GC-MS) were utilized to comprehensively analyze the volatile organic components (VOCs) of three flowers. Meanwhile, a principal component analysis (PCA) and heatmap were applied to distinguish the VOCs identified in PGF, PQF, and PNF. A random forest (RF) analysis was used to screen key factors affecting the discrimination. As a result, 39, 68, and 78 VOCs were identified in three flowers using fast GC e-nose, HS-GC-IMS, and HS-SPME-GC-MS. Nine VOCs were selected as potential chemical markers based on a model of RF for distinguishing these three species. Conclusively, a complete VOC analysis strategy was created to provide a methodological reference for the rapid, simple, and environmentally friendly detection and identification of food products (tea, oil, honey, etc.) and herbs with flavor characteristics and to provide a basis for further specification of their quality and base sources.

## 1. Introduction

Multiple species from the *Panax* genus are known to exhibit tonic effects on human health, such as *Panax ginseng* C.A. Meyer, *Panax quinquefolius* L., and *Panax notoginseng* (Burk.) F.H. Chen are the most widely known and share a large market as drugs, dietary supplements, and foods. Previous research has found that versatile primary and secondary metabolites, including saponins, polysaccharides, flavonoids, amino acids, organic acids, and sterols, etc., are present in these plants. Among these, saponins are the primary bioactive components, exhibiting pharmacological effects on the central nervous system, cardiovascular system, and immune system [1,2]. With the development of modern detection technology, active ingredients similar to those in the respective rhizomes and roots have been found in their flower parts [3,4]. Flower buds are increasingly attracting attention due to their specific health-promoting properties and potential medicinal uses. The flower buds of three *Panax* species (PGF: *P. ginseng*; PQF: *P. quinquefolius*; PNF: *P. notoginseng*) are currently being developed as a new food ingredient, such as in the form of health tea, which is particularly popular in China due to its unique aroma and health benefits. With further development, these flower buds have also been used in beverages and even added to shampoos [5]. There is a significant price difference among the three flowers, each varying in edibility and taste. PGF and PNF have the best reputation and are priced higher than PQF or other ginseng species. However, the flower buds of PGF, PQF, and PNF have similar appearances. Illegal merchants have substituted cheap PQF, which has a similar appearance, for the more expensive PNF or PGF in order to seek exorbitant profits, resulting in a particularly serious issue with these three flowers being mixed in the market [6]. There is an urgent need for a fast and simple method to distinguish these three flowers in order to maintain the stability of the market. Meanwhile, most research on the components in the three flowers has focused on saponins, while studies on the VOCs of these three flowers are hitherto quite limited. Therefore, it was necessary to characterize the VOCs of the three flowers and provide a reference for their product development.

Conventional approaches for plant identification include microscopic examination, the analysis of physicochemical properties, and thin-layer chromatography (TLC) [7]. These conventional methods require professional personnel and are subject to strong objectivity and low accuracy [8]. Modern identification methods mainly rely on instruments for identification, such as high-performance liquid chromatography (HPLC), mass spectrometry, spectrometry, etc. These technologies have become particularly popular in identification due to their advantages of objectivity and accuracy, especially mass spectrometry [9]. Mass spectrometry mainly includes gas chromatography–mass spectrometry (GC-MS) and liquid chromatography–mass spectrometry (LC-MS). Although LC-MS has the characteristic of high sensitivity, it requires complex sample processing in the early stages. GC-MS includes two-dimensional gas chromatography (GC-GC), gas chromatography–mass spectrometry, electronic noses (e-noses), and gas chromatography–olfactometry–mass spectrometry (GC-O-MS). GC-MS and e-noses are widely used in identification due to their high speed and sensitivity [10,11]. Furthermore, the introduction of headspace solid-phase microextraction (HS-SPME) with GC-MS has significantly simplified the processing of complex samples. This innovation offers the benefits of nondestructive sample processing and improved detection efficiency [12]. Similarly to GC-MS, e-nose technology is a mature technology that is widely used in the food industry [13]. It can simulate human olfactory function and has the advantages of convenience and speed. GC-MS and e-noses are particularly advantageous in the determination of medium-to-large VOCs, although their effectiveness may not be as high as that of HS-GC-IMS for small molecules. HS-GC-IMS is an emerging technique with high molecular specificity, sensitivity, easy operation, affordability and nondestructive analysis, making it well-suited for detecting small-molecular-weight VOCs [14]. HS-SPME-GC-MS and HS-GC-IMS are able to promote detection efficiency and accuracy and implement the comprehensive characterization of VOCs [15]. To the best of our knowledge, no research has been conducted on the identification of PGF, PQF, and PNF and the characterization of their VOCs combining HS-SPME-GC-MS, fast GC e-nose, and HS-GC-IMS.

**Figure 1:**
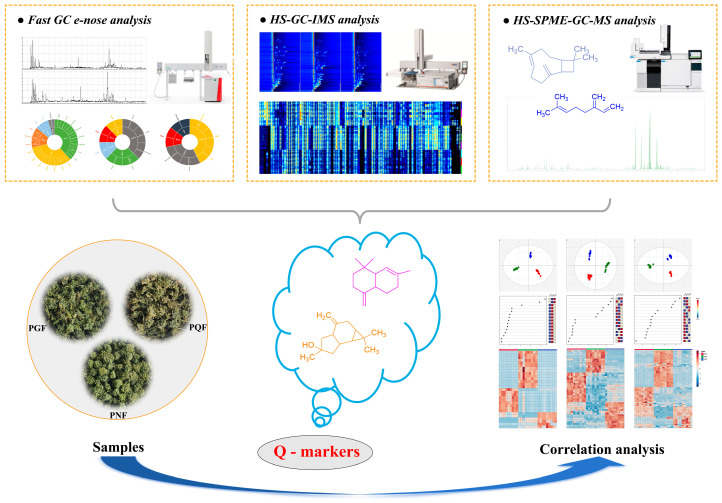
The overall strategy of the experiment.

Therefore, the aim of this study was to establish a complete analytical strategy to achieve a comprehensive characterization and evaluate the flavor characteristics of the VOCs of PGF, PQF, and PNF using HS-SPME-GC-MS, a fast GC e-nose, and HS-GC-IMS combined with multivariate statistical analysis. The differences in the VOCs are further discussed to identify the key markers responsible for these differences. This study aims to achieve the rapid identification of varieties, which can serve as a reference for the establishment of future technologies for the rapid detection and identification of food products and medicinal materials with distinct flavor characteristics as well as for quality control throughout production and circulation. The overall strategy of the experiment is shown in Figure 1.

## 2. Results and Discussion

### 2.1. Characterization of the Flavor Components by Fast GC E-Nose

**Figure 2:**
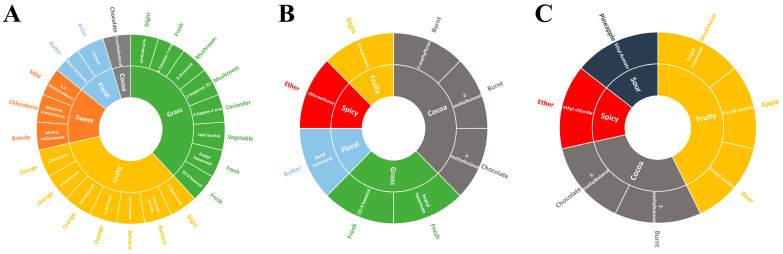
Flavor wheel of the flower buds of (**A**) PNF, (**B**) PQF, and (**C**) PGF based on the fast GC e-nose.

The fast GC e-nose is an advanced olfactive tool with the high-efficiency separation ability of GC and the biological simulation of smell that has been widely used in the food industry [16,17]. Moreover, due to its advantages in the rapid and objective evaluation of the quality of food and medicinal materials, it has been widely developed in the field of identification and separation of food and medicinal materials [18]. In this study, 27 batches of PGF, PQF, and PNF flavor compounds from different regions were detected using two columns with different polarities (MXT-5 and MXT-1701). From the TIC diagram (Figure S1), it can be seen that columns with different polarities have different effects on the separation and detection of the same sample’s odor, mainly in terms of peak intensities. Since the VOCs of flower samples are mostly non-polar compounds, the MXT-5 column was used as the main identification column, while the MXT-1701 column was used as the auxiliary identification column. The calculated Kovats retention indices were matched against the Arochemical base database and a total of 39 compounds were identified from the three flowers. Table 1 presents the relative information of the aroma components. From the flowers of PQF, 11 components were identified, including 4 hydrocarbons, 1 ether, 5 esters, 2 aldehydes, and 2 terpenes. From the flowers of PGF, 14 compounds were identified, including 2 hydrocarbons, 1 ether, 2 esters, 3 aldehydes, 1 terpene, 1 ketone, and 1 furan. From the flowers of PNF, 26 compounds were identified, including 6 hydrocarbons, 5 esters, 4 aldehydes, 2 alcohols, 4 ketones and 5 terpenes. In addition, 3-methylbutanal and α-himachalene were identified as common components in the three flowers. In addition, the sensory characteristics of the three flower flavor components were obtained in the ArochemBase database and their respective flavor wheels were plotted as shown in Figure 2.

The scent characteristics of the three flowers were roughly divided into four types, namely grass, fruit, sweet, flower, and cocoa. PNF had a strong fruity and grassy flavor (Figure 2A). Among the detected aroma components from PNF, seven and eight components were divided into the perception category of fruity flavor and the perception category of grassy flavor, respectively. Fruit aroma components comprised 3-pentanone, leaf acetate, γ-terpinene, α-selinene, tetradecanal, etc.; among the seven fruit aroma components, four were further specific to sweet orange smell. The contents of (*Z*)-3-hexenal and pentadecane in grass flavor were higher, while the contents of (*E*)-2-heptenal and 3-octanone were synergically manifested as light mushroom odor. PGF and PQF had less sweetness and more cocoa flavor, and both had relatively high contents of chloroethane, a spicy flavor component (Figure 2B,C). Therefore, PNF tasted better when used as a flower tea than PQF and PGF.

### 2.2. Qualitative Analysis of the VOCs by HS-GC-IMS

**Figure 3:**
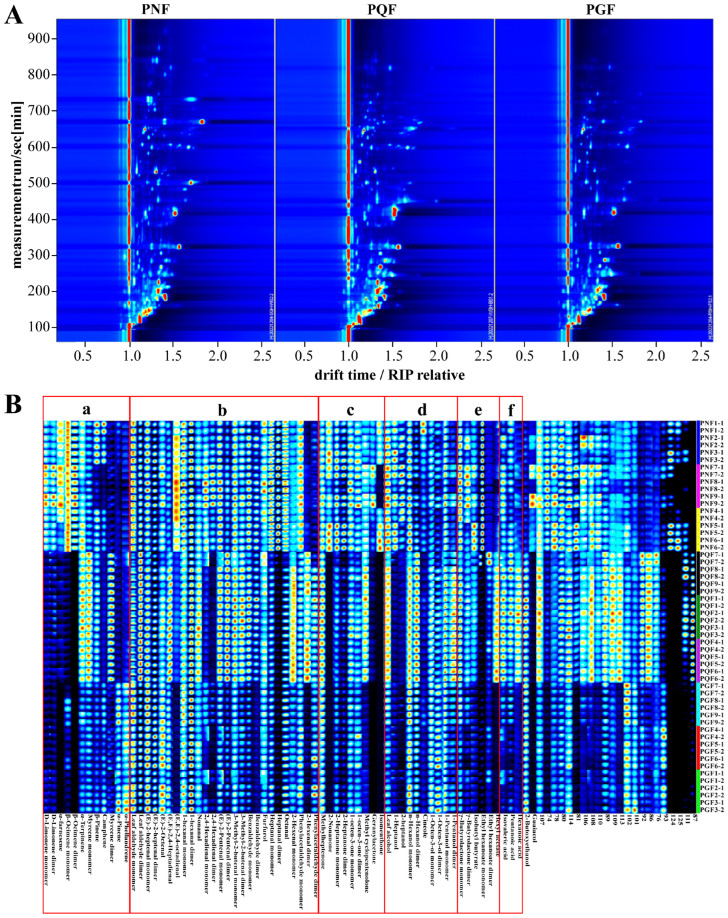
(**A**) Two-dimensional chromatogram results of volatile fractional compositions in the flower bud of PGF, PQF, and PNF. (**B**) The VOC fingerprint of the flower buds of PGF, PQF, and PNF.

HS-GC-IMS as an emerging technique for gas analysis is widely used in the food, medical, and environmental fields because of its high efficiency, non-destructive nature of the sample, fast detection speed, and high information content [19]. In order to achieve the rapid identification of PGF, PQF, and PNF, the VOCs of the three flowers were analyzed via HS-GC-IMS as an untargeted analytical strategy in the present study. The VOCs were identified via Rt and Dt using GC × IMS Library Search. The information of the samples analyzed via HS-GC-IMS for the three types of flowers is shown in the form of topographic maps and fingerprints in Figure 3. A top view of the GC-IMS 3D-topographic plot in PGF, PQF, and PNF samples from different areas is shown in Figure 3A. It can be seen that most of the signals appeared in the retention time of 100–900 s and the drift time of 1.0–2.0 s. Each point in the topographic maps represents a VOC in the sample, and the color shades of the point indicate the relative content of the compound; the redder the color, the higher the relative content, and the bluer the color, the lower the relative content. In Figure 3A, the three flowers differed in composition, and even for the components shared by the three flowers, the relative contents were different. In total, 68 VOCs were identified in the three flowers, including 42 from PGF, 48 from PQF, and 58 from PNF.

To further facilitate a clearer comparison of the VOCs among the three flowers, the chemical composition of the samples was classified and illustrated as fingerprints. The differences of the three flowers in terms of constituent species and content are visually demonstrated in Figure 3B. A total of 68 VOCs were identified for the three flowers, including 12 terpenes, 26 aldehydes, 9 ketones, 10 alcohols, 6 esters, and 3 acids (Table 2), which corresponded to the 6 regions of a, b, c, d, e, and f in Figure 3B, respectively. In addition, there were 22 detected constituents that had not yet been identified. PNF, PGF, and PQF were the most abundant in aldehyde components, with (*E*,*E*)-2,4-octadienal, 2,4-hexadienal dimer, and heptanal dimer being the aldehydes specific to PNF in the three flowers, and 2-hexenal dimer being the aldehyde specific to PQF in the three flowers (Figure 3B-a). Terpenoids were the second most abundant components in the three flowers, and their composition was more different. d-limonene monomer, d-limonene dimer, α-farnesene, β-ocimene monomer, and β-ocimene dimer were the unique terpenoid components of PNF in the three kinds of flowers. Limonene, with some anti-inflammatory and antioxidant effects, has long been used in the food and cosmetics industry. α-farnesene has a strong floral aroma and is often used as an additive in daily chemical products. Myrcene dimer is a unique terpenoid component of PQF in the three flowers. α-pinene and α-phellandrene were the specific terpenoids of PGF in the three flowers (Figure 3B-b). The abundance of ketone constituents was higher in PNF than in PGF and PQF. We found that 2-Nonanone, geraniolactone and isomenthone could be distinguished from the other two flower species as characteristic components of PNF. Methyl cyclopentenolone was higher in PQF (Figure 3B-c). The alcohol composition of the three flowers was more similar, with 1-heptanol and 2-heptanol being higher in PNF, and 1-pentanol monomer and 1-pentanol dimer being higher in PQF (Figure 3B-d). Esters were relatively few in PGF, and ethyl hexanoate monomer and hexyl acetate can be used as the signature ester components of PNF and PQF, respectively (Figure 3B-e). Acids were predominantly found in PQF, such as isovaleric acid, pentanoic acid, and hexanoic acid (Figure 3B-f). In addition, there were many components of PNF and PQF with high content that have not yet been identified.

### 2.3. Qualitative VOCs via HS-SPME-GC-MS

HS-SPME-GC-MS is widely used for VOC detection because of its simplicity, rapidity, and specificity [20]. Moreover, the introduction of solid-phase microextraction (SPME) into GC-MS greatly improves the detection speed and realizes the non-destructive operation of samples [12]. In contrast to the detection of small molecules via GC e-nose and HS-GC-IMS, HS-SPME-GC-MS was able to control the quality of food and medicinal materials from the perspective of large molecules [10]. In this study, the VOCs of three flowers were detected via HS-SPME-GC-MS.

The key parameters were optimized before the experiments formally started. The univariate method was used to select the SPME conditions for each factor individually: three fiber coatings were tested, including polydimethylsiloxane 100 mm phase thickness (PDMS), polydimethylsiloxane/divinylbenzene 65 mm phase thickness (PDMS/DVB), and polydimet-hylsiloxane/carboxen/divinyl benzene 50/30 mm phase thickness (PDMS/CAR/DVB). After filtering the miscellaneous peaks, we compared the number of peaks under different conditions. The number of PDMS peaks was the least and the number of PDMS/DVB peaks was the most. In addition, the incubation temperatures (50 °C, 60 °C, 70 °C) and incubation times (5 min, 10 min, 15 min) were optimized. An analysis of chromatograms showed that the peak area and number of peaks decreased significantly with increasing incubation temperature. Both peak area and peak number were optimal when the incubation temperature was 50 °C. The incubation time was positively correlated with the peak areas of the components, and when the incubation time was 5 min, the peak areas of most of the components were at a suitable level for measurement. Finally, the extraction time was optimized. The samples were extracted for 10 min, 20 min, and 30 min then contrasted with the TIC. A high peak area was achieved for all components at an extraction time of 10 min and the number of peaks did not change significantly with increasing extraction time. Thus, 10 min of extraction time was chosen as the most suitable. The resulted optimal extraction parameters were determined as follows, and the PDMS/DVB coating was chosen and incubated for 5 min at 50 °C, followed by 10 min in an extract.

**Figure 4:**
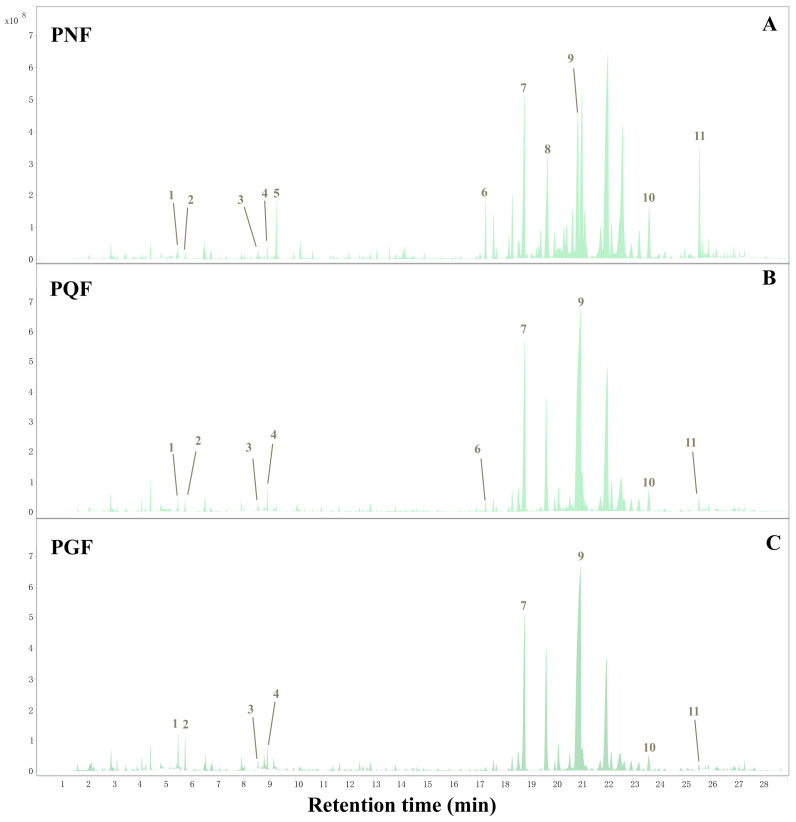
The total ion chromatogram (TLC) of the flower buds of (**A**) PNF, (**B**) PQF, and (**C**) PGF based on HS-SPME-GC-MS.

The TICs of the three different flower samples are shown in Figure 4. The three flowers were overall somewhat similar, but there were some differences in the type and content of VOCs. Qualitatively, a total of 78 VOCs were identified from PQF, PGF, and PNF according to the retention index and matching value, as well as comparison with the literature reference (Table 3), with 66 in PGF, 63 in PQF, and 69 in PNF, and 55 common components among the three. Eleven compounds passed the standard substance verification and are highlighted as numbers in the TIC. All the identified components included 38 terpenes, 12 alcohols, 9 esters, 7 aldehydes, 6 ketones, 3 acids, and 3 others. The results show that the major VOCs in the PQF and PGF samples are relatively similar, mainly including β-elemene, santalene, α-bergamotene, (*E*)-β-farnesene, bicyclosesquiphellandrene, β-selinene, and eremophilene, whereas the major VOCs in the PNF are different and consist mainly of octanal, (−)-isoledene, β-elemene, β-caryophyllene, (−)-aristolene, (*E*)-β-farnesene, valerena-4, 7(11)-diene, β-santalene, germacrene D, bicyclosesquiphellandrene, (−)-α-muurolene, (+)-δ-cadinene, and spathulenol. Spathulenol and β-caryophyllene are important active components of the three species present only in PGF and their relative content was higher than 5%; both exhibit high antioxidant activity and antiproliferative effects, in addition to the anti-inflammatory as well as anti-mycobacterial activity of spathulenol [21,22].

### 2.4. Comprehensive Analysis

**Figure 5:**
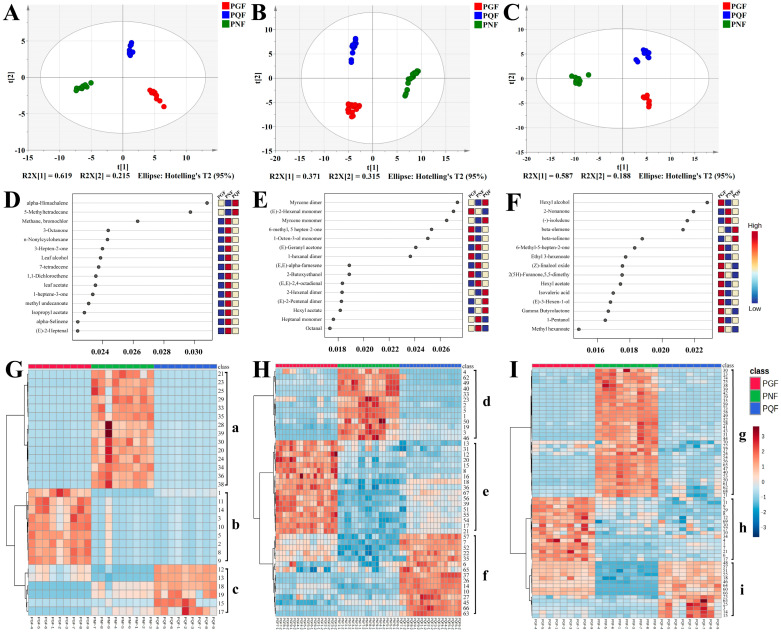
(**A**–**C**): PCA analysis via fast GC e-nose, HS-GC-IMS, and HS-SPME-GC-MS, respectively; (**D**–**F**): RFA via fast GC e-nose, HS-GC-IMS, and HS-SPME-GC-MS, respectively; (**G**–**I**): the heatmap clustering of the VOCs via fast GC e-nose, HS-GC-IMS, and HS-SPME-GC-MS, respectively (The codes of the compounds correspond to those in Table 3).

In order to determine whether fast GC e-nose, HS-GC-IMS, and HS-SPME-GC-MS could distinguish between PGF, PQF, and PNF, the three sets of data collected were statistically analyzed by peak area. Unsupervised PCA was a common method of dimensionality reduction of image processing and the data were visualized [23,24]. It reduced the dimensionality of the data by projecting its variables onto the main factors, thereby providing a visual representation of group clustering trends. Therefore, PCA methods were used for data processing to analyze the differences in chemical composition between all samples. A, B, and C in Figure 5 represent the results of fast GC e-nose analysis, HS-GC-IMS analysis, and HS-SPME-GC-MS analysis, respectively. R^2^X and Q^2^ could evaluate the explanatory and predictive abilities of the models; the closer R^2^X and Q^2^ were to 1, the better the fitness of the model was [25]. The model parameters of fast GC e-nose analysis (R^2^X = 0.922 and Q^2^ = 0.780) show that 92.2% and 78.0% of the total variation could be explained and predicted, respectively. The model parameters of HS-GC-IMS analysis (R^2^X = 0.937 and Q^2^ = 0.840) indicate that 93.7% and 84.0% of the total variation could be explained and predicted, respectively. The model parameters of HS-SPME-GC-MS analysis (R^2^X = 0.834 and Q^2^ = 0.762) indicate that 83.4% and 76.2% of the total variation could be explained and predicted, respectively. The three analysis methods could cluster the three flowers into three categories, which indicates that there were significant differences in the volatile components among the three types of flowers.

The random forest algorithm (RFA) is a powerful and flexible integrated learning algorithm that relies on the result of random combinations of multiple decision tree predictions to improve the accuracy and stability of the model, and it is suitable for regression and classification [26]. The classification trees were set as 2000 in this study. During tree building, one-third of the samples were used as training data and the remaining samples as test samples to obtain an unbiased assessment of the out-of-bag (OOB) error. After several trees, the cumulative OOB error rates decreased to zero in the three types of flowers. Figure 5D–F shows the significant features in the random forest classification model. Fast GC e-nose analysis selected characteristic components with an RFA score value higher than 0.028 for analysis (Figure 5D), and 5-methyltetradecane and α-himachalene were the characteristic components of PQF. The characteristic components with an RFA score value higher than 0.026 were selected for analysis in HS-GC-IMS analysis (Figure 5E); the (*E*)-2-hexenal monomer was most specific in PGF, and the myrcene monomer and myrcene dimer were the characteristic components of PQF. The characteristic components with an RFA score value higher than 0.020 were selected for analysis in HS-SPME-GC-MS analysis (Figure 5F): hexyl alcohol was most specific in PGF; β-elemene was the characteristic components of PQF; and 2-nonanone and (−)-isoledene were the characteristic components of PNF. Therefore, nine VOCs (5-methyltetradecane, α-himachalene, (*E*)-2-hexenal monomer, myrcene monomer, myrcene dimer, hexyl alcohol, β-elemene, 2-nonanone, and (−)-isoledene) were selected as the potential chemical markers based on a model of RF.

To further visualize the differences in VOC matter content among the three types of flowers, MetaboAnalyst 5.0 was used for heat map hierarchical clustering analysis. With the relative content of components identified via fast GC e-nose, HS-GC-IMS, and HS-SPME-GC-MS as variables, each variable was normalized, and the three kinds of flowers were clustered into heatmaps by using Euclidean distance for similarity measure and Ward clustering algorithm. As shown in Figure 5G–I, the components of class a, d and g were more abundant in PNF, the components of class b, e and h were more abundant in PGF, and the components of class c, f, and i were more abundant in PQF. In conclusion, the thermograms formed by clustering the analyzed data from all three instruments were able to demonstrate the differences in the content of the three flowers well.

## 3. Materials and Methods

### 3.1. Sample Source and Preparation

A total of 27 samples belonging to three species, PGF, PQF, and PNF, were purchased in three batches of each species from three different origins. PNF was collected from Wenshan (PNF-1, PNF-2, PNF-3), Qiubei (PNF-4, PNF-5, PNF-6), and Yanshan (PNF-7, PNF-8, PNF-9) all in Yunnan; PGF and PQF were collected from Fusong (PGF-1, PGF-2, PGF-3, PQF-1, PQF-2, PQF-3) and Baishan (PGF-4, PGF-5, PGF-6, PQF-4, PQF-5, PQF-6) all in Jilin, and PGF-7, PGF-8, PGF-9, PQF-7, PQF-8, PQF-9 were from Xinbin, Liaoning Province, China. All samples in the experiments were authenticated by Professor Lijuan Zhang from Tianjin University of Traditional Chinese Medicine. Detailed information about the samples is listed in Supplementary Table S1. The specimens were deposited in the College of Pharmaceutical Engineering of Traditional Chinese Medicine, Tianjin University of Traditional Chinese Medicine, China. All samples were crushed with a grinder and sieved through a 40-mesh sieve. For the subsequent analysis, the powdered sample was immediately stored in an airtight bag in a cool dark, dry room at 20 °C.

### 3.2. Chemicals and Reagents

*N*-alkane C6-C16 standard (Lot: 563121) for fast GC e-nose was purchased from RESTEK (Bellefonte, PA, USA). *N*-ketone C4-C9 standard mix for HS-GC-IMS was purchased from Sinopharm Chemical Reagent Beijing Co., Ltd. (Beijing, China). *N*-alkane C8-C20 standard for HS-SPME-GC-MS was purchased from Sigma-Aldrich Chemical Co., Ltd. (St. Louis, MO, USA). Reference compounds were purchased for identification. Spathulenol (CAS: 6750-60-3, 93%), δ-elemene (CAS: 20307-84-0, 95%), β-elemene (CAS: 515-13-9, 98%), (*E*)-β-farnesene (CAS: 18794-84-8, 80%), 1-octen-3-ol (CAS: 3391-86-4, 98%), (*E*)-3-hexen-1-ol (CAS: 928-97-2, 97%), β-caryophyllene (CAS: 87-44-5, 98%), myrcene (CAS: 123-35-3, 98%), (+)-δ-cadinene (CAS: 483-76-1, 95%) and hexyl alcohol (CAS: 111-27-3, 99%) were bought from Shanghai Yuanye Bio-Technology Co., Ltd. (Shanghai, China). Octanal (CAS: 124-13-0, 97%) was provided by Aladdin Biochemical Technology Co., Ltd. (Shanghai, China).

### 3.3. Fast GC E-Nose Analysis Conditions

The Heracles NEO e-nose (Alpha M.O.S., Toulouse, France) combines the functionality of gas chromatography technology with the pattern recognition technology of an e-nose and is equipped with a headspace autosampler (PAL-RSI, Alpha m.o.s., Toulouse, France) and two different polarity columns (MXT-5: a nonpolar column, 10 m × 0.18 mm, 0.4 µm, with 14% cyanobenzenyl and 86% methylpolysiloxane; MXT-1701: a low-polar column, 10 m × 0.18 mm, 0.4 µm, containing 5% diphenyl, 95% methyl polysiloxane). These two parallel metal capillary ultra-fast columns of different polarities were combined with a flame ionization detector (Fid) and a built-in pre-concentration trap system to dramatically increase detection sensitivity.

Before analysis, 0.5 g of dried powder was added to a 20 mL specialized vial for headspace extraction. To allow the odor to saturate the headspace bottles, the incubation temperature was set to 70 °C for 20 min, with a stirring speed of 500 rpm. At a constant inlet temperature (200 °C) and inlet pressure (10 kPa), 5000 µL of headspace phase was injected into the gas chromatography port using an autosampler at a speed of 125 µL/s. The injector temperature was 200 °C. The analytes were collected in a trap within 229 s from the initial temperature 50 °C to the final 240 °C. The column temperature was initially of 50 °C, then increased to 72 °C at a rate of 0.3 °C per second, then increased to 140 °C at a rate of 3 °C per second, then increased to 190 °C at a rate of 0.7 °C per second, then increased to 200 °C at a rate of 0.3 °C per second, and finally increased to 250 °C at 3 °C per second, remaining at 250 °C for another 10 s. The two FIDs temperatures and gains were set to 260 °C and 12, respectively. Hydrogen was used as the carrier gas at a constant flow of 1.0 mL/min. Each sample was repeatedly measured three times, following the above conditions. The *N*-alkane C6-C16 standard was used for calibration to convert the retention time (RT) into the retention index (RI) of each compound as external references.

### 3.4. HS-GC-IMS Analysis Conditions

The VOCs in the three flowers were analyzed using the HS-GC-IMS system (FlavourSpecÒ, Gesellschaft fur Analytische Sensorsysteme mbH, Dortmund, Germany), which was equipped with an autosampler unit (CTC Analytics AG, Zwingen, Switzerland) and an MXT-5 capillary column (15 m × 0.53 mm ID, 1 µm, CS-Chromatographie Service GmbH, Langerwehe, Germany) [10]. In brief, a 0.5 g powder sample was accurately placed into a 20 mL headspace glass sampling bottle and incubated at 80 °C for 20 min at 500 rpm. After incubation, the headspace samples (500 µL) were automatically injected into the syringe (60 °C) in the splitless mode and then driven into a capillary column of 80 °C isothermal conditions through nitrogen of 99.999% purity; its flow rate was first set at 2 mL/min for 2 min, then increased to 4 mL/min with 7 min, increased to 10 mL/min over 10 min, increased to 100 mL/min over 20 min, increased to 150 mL/min over 30 min, and then was maintained until 45 min. The pre-separated compounds were driven into an ionization chamber and ionized by a 3H ionization source with 300 MBq activity in the positive ion mode. The resulting ions were driven to a drift tube (9.8 cm in length), which was operated on a constant temperature (45 °C) and voltage (5 kV). The flow rate of the drift gas (nitrogen gas) was set at 150 mL/min. Each sample was repeatedly measured three times, following the above conditions. The *N*-ketones C4-C9 standard was used to calculate the RI and drift time (Dt) of each compound as external references.

### 3.5. HS-SPME-GC-MS Analysis Conditions

For HS-SPME-GC-MS, we accomplished headspace autosampling by installing the SPME fiber (Supelco, Bellefonte, PA, UAS) on a MultiPurpose sampler (Gerstel, Mülheim, Germany) in conjunction with a GC autosampling system equipped with an Agilent 7890B-7000D gas chromatography and mass spectrometry detector (Agilent, Santa Clara, CA, USA; Thermo Fisher, Waltham, MA, USA). The GC was fitted with a HP-5MS elastic quartz capillary column (30 m × 0.25 mm × 0.25 µm, 19091S-433, J&W Scientific, Folsom, CA, USA) [27]. Firstly, 0.1 g of sample was weighed accurately into a 20 mL headspace glass sampling bottle (Zhejiang HAMAG Technology, Ningbo, China), then the headspace bottle was sealed with a screw cap with a silicon gasket. The sample was incubated at 50 °C incubation temperature for 5 min and then the SPME needle was inserted into the headspace glass sampling bottle for extraction for 10 min. Subsequently, the SPME needle immediately plugged into the heated injection port at desorption for 5 min (250 °C, splitless mode). The flow rate of helium (>99.999%) as the carrier gas was 1 mL/min. The GC column temperature was programmed as follows: initially programmed at 40 °C for 2 min, then changed at a rate of 16 °C per minute to 60 °C, at a rate of 4 °C per minute to 99 °C, at a rate of 34 °C per minute to 133 °C, then rose to 134 °C at a rate of 0.5 °C per minute, then at a rate of 2 °C per minute to 134 °C, at a rate of 0.5 °C per minute to 136 °C, at a rate of 2 °C per minute to 141 °C; eventually, the temperature changed at a rate of 22 °C per minute to 240 °C. The GC total running time was 29 min. The MS was operated in electron ionization (EI) mode at an ionizing energy of 70 eV. The injection port and ion source temperature were set at 250 °C and 230 °C, respectively. The quadrupole temperature was 150 °C. The mass spectra (MS1 full scan mode) were scanned from 50 to 600 Da. All samples were prepared in duplicate for analysis. An *N*-alkane C8–C20 standard was used to calculate the RI of each compound as external references.

### 3.6. Data Analysis

The data acquisition and processing of fast GC e-nose was performed using Alpha software 2021 (version 7.2.8, Alpha Software Co., Burlington, MA, USA). The VOCs detected via fast GC e-nose were identified based on the Kovats retention index compared with the AroChemBase professional flavor database qualitatively. The extraction and analysis of the HS-GC-IMS data were performed with a Laboratory Analytical Viewer (LAV) (version 2.2.1, G.A.S, Dortmund, Germany). VOCs detected via HS-GC-IMS were identified based on the calculated RI and drift time (drift time, Dt) compared with the database of IMS, and the fingerprint was established using a gallery plot. VOCs detected via HS-SPME-GC-MS were identified from the standard NIST17 library (matching degree > 750, RI) and reference compounds [27]. SIMCA14.1 was used to build the model of principal component analysis (PCA). A heat map and random forest (RF) model were performed using the online website MetaboAnalyst 5.0 for data processing.

## 4. Conclusions

In summary, this research constructed a method for identifying and characterizing PQF, PGF, and PNF based on fast GC e-nose, HS-GC-IMS, and HS-SPME-GC-MS combined with multivariate statistics. The fingerprint, flavor wheel, and multivariate statistical results could effectively visualize the characteristics of the three flowers. The results indicate that the three flowers could be accurately and objectively distinguished without relying on appearance features. This method provides valuable technology for the authenticity and quality control of food products and medicinal materials with flavor characteristics. Fast GC e-nose and HS-GC-IMS provided an objective method for odor identification due to their fast and easy-to-use advantages. HS-SPME-GC-MS revealed the differences in chemical characteristics of VOCs among the three flowers (PQF, PGF, and PNF), providing scientific reference for the development of their health flower tea. Meanwhile, nine components (5-methyltetradecane, α-himachalene, (*E*)-2-hexenal monomer, myrcene monomer, myrcene dimer, hexyl alcohol, β-elemene, 2-nonanone, and (−)-isoledene) were identified through multivariate statistical analysis as chemical markers for distinguishing these three species. The proposed method is fast, simple, environmentally friendly, and could successfully distinguish PQF, PGF, and PNF using scent.

## Figures and Tables

**Table 1:** The types, relative content, and sensory description of aroma components in the flower bud of PGF, PQF, and PNF based on the RI on two columns (MXT-5 and MXT-1701) via fast GC e-nose.

NO.	Compounds	Formula	CAS	MXT-5		MXT-1701		Relative Content (%)			Sensory Description
				RT_1_ (s)	RI_1_	RT_2_ (s)	RI_2_	PGF	PQF	PNF	
1	chloroethane	C_2_H_5_Cl	75-00-3	16.95	450	20.17	521	39.75	12.57	---	Spicy; Ether
2	2-methylpentane	C_6_H_14_	107-83-5	21.49	544	21.67	553	4.56	---	---	---
3	3-methylpentane	C_6_H_14_	96-14-0	23.45	585	22.30	566	3.29	---	---	---
4	diisopropyl ether	C_6_H_14_O	108-20-3	24.16	599	25.09	613	4.90	4.06	---	---
5	ethyl acetate	C_4_H_8_O_2_	141-78-6	25.72	612	29.49	665	3.32	---	---	Sour; Pineapple
6	3-methylbutanal	C_5_H_10_O	590-86-3	30.43	650	37.19	725	6.57	7.33	5.57	Cocoa; Chocolate
7	2-methylbutanal	C_5_H_10_O	96-17-3	31.73	661	38.22	731	4.51	4.74	---	Cocoa; Burnt
8	propyl acetate	C_5_H_10_O_2_	109-60-4	39.20	711	45.60	770	1.81	---	---	Fruity; Pear
9	(*E*)-4-octene	C_8_H_16_	14850-23-8	60.12	802	61.57	835	2.61	---	---	---
10	pentyl acetate	C_7_H_14_O_2_	628-63-7	91.97	923	93.48	964	1.36	---	---	Fruity; Apple
11	propyl nonanoate	C_12_H_24_O_2_	6513-03-7	153.69	1411	151.01	1443	3.14	---	---	Fruity; Muskmelon
12	5-methyltetradecane	C_15_H_32_	25117-32-2	157.55	1437	152.85	1456	3.11	6.35	---	---
13	α-himachalene	C_15_H_24_	3853-83-6	160.79	1459	157.41	1490	19.24	41.32	3.48	---
14	decyl acrylate	C_13_H_24_O_2_	2156-96-9	170.14	1521	166.19	1551	1.82	---	---	---
15	2-methylfuran	C_5_H_6_O	594-20-7	24.21	600	26.17	626	---	1.30	---	Cocoa; Burnt
16	3-pentanone	C_5_H_10_O	96-22-0	36.55	700	43.34	758	---	2.02	3.36	Fruity; Slight
17	(*Z*)-3-hexenal	C_6_H_10_O	6789-80-6	60.05	802	73.62	877	---	10.26	3.94	Grass; Fresh
18	butyl octanoate	C_12_H_24_O_2_	589-75-3	153.73	1411	151.05	1443	---	7.16	3.72	Floral; Butter
19	heptyl hexanoate	C_13_H_26_O_2_	6976-72-3	163.15	1475	162.66	1527	---	2.89	1.70	Grass; Fresh
20	1, 1-dichloroethene	C_2_H_2_Cl_2_	75-35-4	20.75	529	21.54	550	---	---	7.35	Sweet; Mild
21	hexane	C_6_H_14_	110-54-3	24.03	597	23.34	587	---	---	7.43	Gasoline
22	methane, bromochloro-	CH_2_BrCl	74-97-5	26.71	620	31.95	694	---	---	2.12	Sweet; Chloroform
23	isopropyl acetate	C_5_H_10_O_2_	108-21-4	29.74	645	36.53	722	---	---	7.49	Banana; Fruity
24	1-heptene-3-one	C_7_H_12_O	2918-13-0	82.43	879	92.00	968	---	---	0.79	Metal
25	leaf alcohol	C_7_H_12_O_2_	33467-73-1	91.90	923	94.75	985	---	---	1.29	Grass; Vegetable
26	3-hepten-2-one	C_7_H_12_O	1119-44-4	95.12	946	103.26	1049	---	---	0.44	Grass; Coriander
27	(*E*)-2-heptenal	C_7_H_12_O	18829-55-5	97.93	966	104.37	1059	---	---	0.75	Grass; Mushroom
28	3-octanone	C_8_H_16_O	106-68-3	100.54	984	105.74	1070	---	---	0.57	Grass; Mushroom
29	leaf acetate	C_8_H_14_O_2_	3681-71-8	103.39	1005	107.51	1085	---	---	5.33	Banana; Fruity
30	γ-Terpinene	C_10_H_16_	99-85-4	108.54	1051	111.21	1117	---	---	1.17	Orange; Fruity
31	*p*-cymenene	C_10_H_12_	1195-32-0	113.43	1094	114.63	1189	---	---	0.48	Orange; Fruity
32	linalool	C_10_H_18_O	78-70-6	114.85	1107	120.14	1196	---	---	0.34	Floral; Anise
33	7-tetradecene	C_14_H_28_	41446-63-3	152.13	1400	147.50	1417	---	---	1.83	Grass; Fresh
34	methyl undecanoate	C_12_H_24_O_2_	1731-86-8	157.63	1438	154.77	1470	---	---	1.86	Brandy; Sweet
35	3-ethyltridecane	C_15_H_32_	13286-73-2	160.82	1459	157.49	1490	---	---	12.07	---
36	pentadecane	C_15_H_32_	629-62-9	163.07	1474	158.29	1496	---	---	7.53	Grass; Sligh
37	α-selinene	C_15_H_24_	473-13-2	168.05	1507	162.66	1527	---	---	3.80	Fruity; Orange
38	*n*-nonylcyclohexane	C_15_H_30_	2883-02-5	173.23	1540	168.80	1569	---	---	3.08	---
39	tetradecanal	C_14_H_28_O	124-25-4	183.71	1606	191.03	1723	---	---	3.34	Fruity; Orange

Note: RT_1_: retention time in column 1 (MXT-5); RI_1_: retention index for compounds from column 1; RT_2_: retention time in column 2 (MXT-1701); RI_2_: retention index for compounds from column 2; “---” means undetected.

**Table 2:** Identification of the VOCs in the flower buds of PGF, PQF, and PNF via HS-GC-IMS.

NO.	Compounds	CAS	Formula	RI	RT	DT
	Terpenoids (12)					
1	d-limonene monomer	138-86-3	C_10_H_16_	1035.4	719.445	1.22279
2	d-limonene dimer	138-86-3	C_10_H_16_	1035.6	719.874	1.28562
3	α-farnesene	502-61-4	C_15_H_24_	1484.8	1800.242	1.43576
4	β-ocimene monomer	13877-91-3	C_10_H_16_	1044.9	733.618	1.21737
5	β-ocimene dimer	13877-91-3	C_10_H_16_	1044.9	733.618	1.70485
6	α-terpinene	99-86-5	C_10_H_16_	1022.9	701.450	1.22171
7	myrcene monomer	123-35-3	C_10_H_16_	989.8	654.315	1.22072
8	β-pinene	127-91-3	C_10_H_16_	975.0	626.315	1.21973
9	camphene	79-92-5	C_10_H_16_	952.5	586.005	1.21670
10	myrcene dimer	123-35-3	C_10_H_16_	989.7	654.067	1.71957
11	α-pinene	80-56-8	C_10_H_16_	937.1	559.925	1.22059
12	α-phellandrene	99-83-2	C_10_H_16_	1006.6	678.514	1.21871
	Aldehydes (26)					
13	leaf aldehyde monomer	6728-26-3	C_6_H_10_O	853.6	417.340	1.18119
14	leaf aldehyde dimer	6728-26-3	C_6_H_10_O	853.6	417.340	1.51879
15	(*E*)-2-heptenal monomer	18829-55-5	C_7_H_12_O	961.4	601.567	1.25874
16	(*E*)-2-heptenal dimer	18829-55-5	C_7_H_12_O	962.4	603.316	1.67021
17	(*E*)-2-octenal	2548-87-0	C_8_H_14_O	1055.4	749.533	1.33296
18	(*E*,*E*)-2,4-heptadienal	4313-03-5	C_7_H_10_O	1011.1	684.657	1.19462
19	(*E*,*E*)-2,4-octadienal	30361-28-5	C_8_H_12_O	1112.2	841.551	1.26815
20	1-hexanal monomer	66-25-1	C_6_H_12_O	794.9	325.791	1.25660
21	1-hexanal dimer	66-25-1	C_6_H_12_O	794.9	325.791	1.56403
22	nonanal	124-19-6	C_9_H_18_O	1100.5	821.655	1.47236
23	2,4-hexadienal monomer	142-83-6	C_6_H_8_O	914.4	523.595	1.11152
24	2,4-hexadienal dimer	142-83-6	C_6_H_8_O	913.8	522.703	1.43864
25	(*E*)-2-pentenal monomer	1576-87-0	C_5_H_8_O	750.4	269.500	1.10926
26	(*E*)-2-pentenal dimer	1576-87-0	C_5_H_8_O	750.1	269.104	1.36181
27	3-methyl-2-butenal monomer	107-86-8	C_5_H_8_O	781.1	307.234	1.09186
28	3-methyl-2-butenal dimer	107-86-8	C_5_H_8_O	781.1	307.234	1.36333
29	benzaldehyde monomer	100-52-7	C_7_H_6_O	965.1	608.215	1.15227
30	benzaldehyde dimer	100-52-7	C_7_H_6_O	964.7	607.515	1.46975
31	furfurol	98-01-1	C_5_H_4_O_2_	833.9	383.937	1.08606
32	heptanal monomer	111-71-7	C_7_H_14_O	901.7	504.317	1.33085
33	heptanal dimer	111-71-7	C_7_H_14_O	901.4	503.868	1.70253
34	octanal	124-13-0	C_8_H_16_O	1001.6	671.617	1.40190
35	2-hexenal monomer	505-57-7	C_6_H_10_O	846.0	404.049	1.18141
36	phenylacetaldehyde monomer	122-78-1	C_8_H_8_O	1040.1	726.374	1.24860
37	2-hexenal dimer	505-57-7	C_6_H_10_O	845.4	403.120	1.51587
38	phenylacetaldehyde dimer	122-78-1	C_8_H_8_O	1039.4	725.458	1.53261
	Ketones (9)					
39	methylheptenone	110-93-0	C_8_H_14_O	987.2	649.334	1.17553
40	2-nonanone	821-55-6	C_9_H_18_O	1089.4	803.252	1.40756
41	2-heptanone monomer	110-43-0	C_7_H_14_O	889.0	484.585	1.26395
42	2-heptanone dimer	110-43-0	C_7_H_14_O	889.0	484.585	1.63563
43	1-octen-3-one monomer	4312-99-6	C_8_H_14_O	980.1	635.857	1.26833
44	1-octen-3-one dimer	4312-99-6	C_8_H_14_O	980.9	637.257	1.68364
45	methyl cyclopentenolone	80-71-7	C_6_H_8_O_2_	1034.7	718.496	1.16383
46	geranylacetone	3796-70-1	C_13_H_22_O	1454.6	1692.569	1.45301
47	isomenthone	491-07-6	C_10_H_18_O	1132.3	876.787	1.33585
	Alcohols (10)					
48	leaf alcohol	928-96-1	C_6_H_12_O	861.9	432.186	1.23108
49	1-heptanol	111-70-6	C_7_H_16_O	978.5	632.708	1.39685
50	2-heptanol	543-49-7	C_7_H_16_O	917.0	527.636	1.37669
51	*n*-hexanol monomer	111-27-3	C_6_H_14_O	874.8	456.332	1.32838
52	*n*-hexanol dimer	111-27-3	C_6_H_14_O	873.6	454.090	1.63687
53	cineole	470-82-6	C_10_H_18_O	1026.9	707.167	1.29090
54	1-octen-3-ol monomer	3391-86-4	C_8_H_16_O	983.2	641.687	1.15881
55	1-octen-3-ol dimer	3391-86-4	C_8_H_16_O	983.6	642.372	1.59962
56	1-pentanol monomer	71-41-0	C_5_H_12_O	762.5	283.783	1.25661
57	1-pentanol dimer	71-41-0	C_5_H_12_O	760.5	281.329	1.51920
	Esters (6)					
58	γ-butyrolactone monomer	96-48-0	C_4_H_6_O_2_	922.2	535.708	1.08555
59	γ-butyrolactone dimer	96-48-0	C_4_H_6_O_2_	921.3	534.302	1.30269
60	isobutyl butyrate	539-90-2	C_8_H_16_O_2_	958.4	596.249	1.33085
61	ethyl hexanoate monomer	123-66-0	C_8_H_16_O_2_	998.9	667.908	1.33545
62	ethyl hexanoate dimer	123-66-0	C_8_H_16_O_2_	999.6	668.767	1.79910
63	hexyl acetate	142-92-7	C_8_H_16_O_2_	1014.1	688.925	1.38891
	Acids (3)					
64	isovaleric acid	503-74-2	C_5_H_10_O_2_	838.0	390.742	1.22064
65	pentanoic acid	109-52-4	C_5_H_10_O_2_	926.8	543.104	1.50554
66	hexanoic acid	142-62-1	C_6_H_12_O_2_	1006.1	677.786	1.29537
	Others (2)					
67	2-butoxyethanol	111-76-2	C_6_H_14_O_2_	901.7	504.317	1.20820
68	guaiacol	90-05-1	C_7_H_8_O_2_	1074.1	778.631	1.24328

**Table 3:** Volatile chemical components identified in the flower buds of PGF, PQF, and PNF via HS-SPME-GC-MS.

NO.	Compounds	RT	CAS	Formula	Relative Content (%)			Structure Type
		(Min)			PGF	PQF	PNF	
1	1-pentanol	3.887	71-41-0	C_5_H_12_O	0.15 ± 0.02	0.07 ± 0.02	0.04 ± 0.01	Alcohols
2	2, 3-butanediol	4.076	513-85-9	C_4_H_10_O_2_	0.43 ± 0.06	0.16 ± 0.10	0.20 ± 0.07	Alcohols
3	hexanal	4.406	66-25-1	C_6_H_12_O	0.77 ± 0.13	2.16 ± 1.05	0.39 ± 0.06	Aldehydes
4	isovaleric acid	5.354	503-74-2	C_5_H_10_O_2_	0.20 ± 0.11	---	---	Acids
5	hex-2-enal	5.414	505-57-7	C_6_H_10_O	0.17 ± 0.01	0.17 ± 0.06	0.10 ± 0.06	Aldehydes
6	(*E*)-3-hexen-1-ol *	5.474	928-97-2	C_6_H_12_O	1.39 ± 0.14	0.43 ± 0.14	0.36 ± 0.11	Alcohols
7	hexyl alcohol *	5.748	111-27-3	C_6_H_14_O	1.37 ± 0.15	0.51 ± 0.09	0.21 ± 0.04	Alcohols
8	γ-butyrolactone	6.786	96-48-0	C_4_H_6_O_2_	0.54 ± 0.12	---	0.21 ± 0.13	Esters
9	methyl hexanoate	7.081	106-70-7	C_7_H_14_O_2_	0.13 ± 0.03	0.02 ± 0.01	0.05 ± 0.02	Esters
10	α-pinene	7.340	80-56-8	C_10_H_16_	0.12 ± 0.02	0.07 ± 0.02	0.07 ± 0.01	Terpenes
11	2(5*H*)-furanone, 5, 5-dimethy	7.834	20019-64-1	C_6_H_8_O_2_	0.06 ± 0.01	0.01 ± 0.01	0.03 ± 0.01	Esters
12	benzaldehyde	8.034	100-52-7	C_7_H_6_O	0.28 ± 0.04	0.14 ± 0.03	0.16 ± 0.02	Aldehydes
13	1-heptanol	8.298	111-70-6	C_7_H_16_O	0.01 ± 0.01	0.02 ± 0.01	0.09 ± 0.02	Alcohols
14	sabinene	8.413	3387-41-5	C_10_H_16_	0.04 ± 0.01	0.08 ± 0.04	0.02 ± 0.00	Terpenes
15	1-octen-3-ol *	8.558	3391-86-4	C_8_H_16_O	0.43 ± 0.14	1.11 ± 0.49	0.28 ± 0.10	Alcohols
16	hexanoic acid	8.712	142-62-1	C_6_H_12_O_2_	0.25 ± 0.15	0.07 ± 0.09	0.02 ± 0.06	Acids
17	methyl isohexe	8.792	110-93-0	C_8_H_14_O	0.89 ± 0.16	0.28 ± 0.09	0.22 ± 0.17	Ketones
18	myrcene *	8.917	123-35-3	C_10_H_16_	1.05 ± 0.22	1.03 ± 0.15	0.26 ± 0.13	Terpenes
19	octanal *	9.271	124-13-0	C_8_H_16_O	---	---	2.10 ± 0.27	Aldehydes
20	ethyl 3-hexenoate	9.351	2396-83-0	C_8_H_14_O_2_	0.04 ± 0.05	---	---	Esters
21	hexyl acetate	9.586	142-92-7	C_8_H_16_O_2_	0.07 ± 0.02	0.02 ± 0.01	---	Esters
22	*p*-cymene	9.925	99-87-6	C_10_H_14_	0.11 ± 0.04	0.07 ± 0.02	0.16 ± 0.02	Terpenes
23	dipentene	10.065	138-86-3	C_10_H_16_	0.29 ± 0.05	0.37 ± 0.06	0.22 ± 0.17	Terpenes
24	benzyl alcohol	10.185	100-51-6	C_7_H_8_O	0.12 ± 0.10	0.06 ± 0.08	0.48 ± 0.14	Alcohols
25	3-octen-2-one	10.369	1669-44-9	C_8_H_14_O	0.04 ± 0.02	0.42 ± 0.27	0.04 ± 0.01	Ketones
26	lilac lactone	10.424	1073-11-6	C_7_H_10_O_2_	0.01 ± 0.01	---	0.02 ± 0.01	Ketones
27	phenylacetaldehyde	10.524	122-78-1	C_8_H_8_O	0.11 ± 0.02	0.07 ± 0.02	0.06 ± 0.01	Aldehydes
28	trans-β-ocimene	10.664	3779-61-1	C_10_H_16_	0.06 ± 0.02	0.06 ± 0.02	0.26 ± 0.07	Terpenes
29	(*Z*)-linalool oxide	11.457	5989-33-3	C_10_H_18_O_2_	0.23 ± 0.02	0.06 ± 0.02	0.09 ± 0.01	Alcohols
30	heptanoic acid	11.657	111-14-8	C_7_H_14_O_2_	---	---	0.15 ± 0.06	Acids
31	2-nonanone	12.071	821-55-6	C_9_H_18_O	---	0.04 ± 0.01	0.22 ± 0.04	Ketones
32	linalool	12.320	78-70-6	C_10_H_18_O	0.07 ± 0.01	0.03 ± 0.00	0.04 ± 0.01	Alcohols
33	4-acetyl-1-methyl-1-5-cyclohexene	13.299	6090-09-1	C_9_H_14_O	0.06 ± 0.01	0.05 ± 0.02	0.02 ± 0.00	Terpenes
34	2-isobutyl-3-methoxypyrazine	14.252	24683-00-9	C_9_H_14_N_2_O	0.07 ± 0.01	0.04 ± 0.02	---	Others
35	ethyl caprylate	14.521	106-32-1	C_10_H_20_O_2_	---	---	0.08 ± 0.01	Esters
36	safrana	14.596	116-26-7	C_10_H_14_O	0.06 ± 0.01	0.04 ± 0.01	0.05 ± 0.00	Aldehydes
37	β-cyclocitral	14.975	432-25-7	C_10_H_16_O	0.11 ± 0.02	0.08 ± 0.02	0.17 ± 0.02	Aldehydes
38	5-butyldihydro-2(3*H*)-7-furanone	15.614	104-50-7	C_8_H_14_O_2_	---	---	0.06 ± 0.02	Esters
39	bornyl acetate	16.188	76-49-3	C_12_H_20_O_2_	0.02 ± 0.00	0.02 ± 0.01	0.08 ± 0.02	Esters
40	δ-elemene *	17.341	20307-84-0	C_15_H_24_	0.20 ± 0.04	0.20 ± 0.19	1.42 ± 0.19	Terpenes
41	(−)-α-cubebene	17.645	17699-14-8	C_15_H_24_	0.53 ± 0.02	0.57 ± 0.09	1.51 ± 0.19	Terpenes
42	longicyclene	18.159	1137-12-8	C_15_H_24_	---	---	0.12 ± 0.03	Terpenes
43	α-copaene	18.239	3856-25-5	C_15_H_24_	0.17 ± 0.02	0.23 ± 0.07	0.87 ± 0.11	Terpenes
44	(−)-isoledene	18.364	95910-36-4	C_15_H_24_	0.73 ± 0.04	1.06 ± 0.16	2.37 ± 0.39	Terpenes
45	β-bourbonene	18.639	5208-59-3	C_15_H_24_	---	---	0.61 ± 0.50	Terpenes
46	β-elemene *	18.843	515-13-9	C_15_H_24_	12.7 ± 0.17	13.78 ± 0.42	8.16 ± 0.17	Terpenes
47	β-maaliene	19.392	489-29-2	C_15_H_24_	0.02 ± 0.01	0.03 ± 0.04	0.33 ± 0.05	Terpenes
48	santalene	19.682	512-61-8	C_15_H_24_	9.51 ± 0.23	9.50 ± 0.80	---	Terpenes
49	β-caryophyllene *	19.737	87-44-5	C_15_H_24_	---	---	6.27 ± 0.23	Terpenes
50	β-copaene	20.011	18252-44-3	C_15_H_24_	0.58 ± 0.03	0.70 ± 0.13	1.14 ± 0.07	Terpenes
51	α-bergamotene	20.151	17699-05-7	C_15_H_24_	2.05 ± 0.13	2.08 ± 0.26	0.49 ± 0.22	Terpenes
52	aromadendrene	20.211	489-39-4	C_15_H_24_	---	---	0.68 ± 0.05	Terpenes
53	α-guaiene	20.355	3691-12-1	C_15_H_24_	0.06 ± 0.03	0.09 ± 0.12	1.07 ± 0.16	Terpenes
54	α-himachalene	20.460	3853-83-6	C_15_H_24_	0.06 ± 0.08	0.13 ± 0.19	1.61 ± 0.10	Terpenes
55	(+)-epi-β-santalen	20.585	25532-78-9	C_15_H_24_	1.37 ± 0.15	1.35 ± 0.21	0.15 ± 0.09	Terpenes
56	(−)-aristolene	20.680	6831-16-9	C_15_H_24_	0.12 ± 0.11	0.19 ± 0.25	2.23 ± 0.16	Terpenes
57	(*E*)-β-farnesene *	21.004	18794-84-8	C_15_H_24_	37.4 ± 0.98	35.32 ± 3.09	8.30 ± 1.18	Terpenes
58	valerena-4, 7(11)-diene	21.064	351222-66-7	C_15_H_24_	---	---	9.59 ± 0.41	Terpenes
59	β-santalene	21.169	511-59-1	C_15_H_24_	---	---	2.57 ± 0.17	Terpenes
60	cis-muurola-4(14), 5-diene	21.234	157477-72-0	C_15_H_24_	0.38 ± 0.02	0.33 ± 0.09	---	Terpenes
61	germacrene D	21.768	23986-74-5	C_15_H_24_	1.13 ± 0.03	1.34 ± 0.24	2.09 ± 0.16	Terpenes
62	bicyclosesquiphellandrene	22.002	54324-03-7	C_15_H_24_	13.5 ± 0.29	13.45 ± 3.42	18.87 ± 0.93	Terpenes
63	β-selinene	22.192	17066-67-0	C_15_H_24_	1.56 ± 0.05	2.78 ± 0.73	1.63 ± 0.16	Terpenes
64	eremophilene	22.526	10219-75-7	C_15_H_24_	2.81 ± 0.17	3.79 ± 0.88	---	Terpenes
65	(−)-α-muurolene	22.614	10208-80-7	C_15_H_24_	---	---	9.81 ± 1.20	Terpenes
66	α-bulnesene	22.691	3691-11-0	C_15_H_24_	0.94 ± 0.05	0.89 ± 0.15	---	Terpenes
67	β-bisabolene	22.959	495-61-4	C_15_H_24_	0.99 ± 0.05	0.95 ± 0.11	0.82 ± 0.09	Terpenes
68	(+)-δ-cadinene *	23.634	483-76-1	C_15_H_24_	1.31 ± 0.05	1.17 ± 0.37	2.37 ± 0.19	Terpenes
69	dihydroactinidiolide	23.889	17092-92-1	C_11_H_16_O_2_	0.16 ± 0.01	0.09 ± 0.04	0.08 ± 0.03	Esters
70	cubenene	24.038	16728-99-7	C_15_H_24_	0.19 ± 0.01	0.11 ± 0.04	0.24 ± 0.07	Terpenes
71	selina-3, 7(11)-diene	24.243	6813-21-4	C_15_H_24_	0.16 ± 0.01	0.16 ± 0.06	0.27 ± 0.03	Terpenes
72	(−)-spathulenol	25.126	77171-55-2	C_15_H_24_O	0.25 ± 0.03	0.28 ± 0.19	---	Alcohols
73	spathulenol *	25.575	6750-60-3	C_15_H_24_O	0.19 ± 0.07	0.34 ± 0.20	5.29 ± 0.72	Alcohols
74	isoaromadendrene epoxide	25.710	---	---	0.10 ± 0.05	0.13 ± 0.03	0.62 ± 0.15	Others
75	mintketone	25.940	73809-82-2	C_15_H_24_O	0.25 ± 0.04	0.35 ± 0.05	0.74 ± 0.16	Ketones
76	aromadendrene oxide	27.123	---	---	0.21 ± 0.03	0.28 ± 0.05	0.19 ± 0.09	Others
77	4(15), 5, 10(14)-germacratrien-1-ol	27.332	81968-62-9	C_15_H_24_O	0.35 ± 0.05	0.48 ± 0.28	0.47 ± 0.46	Alcohols
78	phytone	28.710	502-69-2	C_18_H_36_O	0.07 ± 0.01	0.07 ± 0.05	0.02 ± 0.01	Ketones

Note: “---” means undetected; * means the compounds identified with the standard reference compound.

## Data Availability

Data are contained within the article and Supplementary Materials.

## Supplementary Materials

The following supporting information can be downloaded at: https://www.mdpi.com/article/10.3390/molecules29030602/s1, Table S1: Details of samples of the flower bud of *P. ginseng* (PGF), *P. quinquefolius*; Figure S1: (A) The total ion chromatogram (TIC) of the flower bud of PGF, PQF, and PNF aroma compounds identified via fast GC e-nose (MXT-5-FID1 and MXT-1701-FID2); Figure S2: Reference compounds spectrogram.
