# Supplementary material for: Comparative Analysis of Volatile Compounds in the Flower Buds of Three Panax Species Using Fast Gas Chromatography Electronic Nose, Headspace-Gas Chromatography-Ion Mobility Spectrometry, and Headspace Solid Phase Microextraction-Gas Chromatography-Mass Spectrometry Coupled with Multivariate Statistical Analysis
Source: Molecules. 2024 Jan 26;29(3):602. doi: 10.3390/molecules29030602 (PMC10856343; doi:10.3390/molecules29030602)
Supplement: Supplementary file 1 [file molecules-29-00602-s001.zip › molecules-2767631-supplementary.pdf]

Supplementary Data

Comparative Analysis of Volatile Compounds in the Flower Buds of Three Panax Species Using Fast GC E-Nose, HS-GC-IMS, and HS-SPME-GC-MS Coupled with Multivariate Statistical Analysis

Table S1. Details of samples of the flower bud of *P. ginseng* (PGF), *P. quinquefolius* (PQF) and *P. notoginseng* (PNF).

NO	Samole Name	Province	City	Batch Code
PGF-1	flower bud of *P. ginseng*	Jilin	Fusong	20220601
PGF-2	flower bud of *P. ginseng*	Jilin	Fusong	20220802
PGF-3	flower bud of *P. ginseng*	Jilin	Fusong	20221002
PGF-4	flower bud of *P. ginseng*	Jilin	Baishan	20220703
PGF-5	flower bud of *P. ginseng*	Jilin	Baishan	20220901
PGF-6	flower bud of *P. ginseng*	Jilin	Baishan	20221008
PGF-7	flower bud of *P. ginseng*	Liaoning	Xinbin	20220806
PGF-8	flower bud of *P. ginseng*	Liaoning	Xinbin	20220913
PGF-9	flower bud of *P. ginseng*	Liaoning	Xinbin	20221011
PQF-1	flower bud of *P. quinquefolius*	Jilin	Fusong	20220606
PQF-2	flower bud of *P. quinquefolius*	Jilin	Fusong	20220809
PQF-3	flower bud of *P. quinquefolius*	Jilin	Fusong	20221008
PQF-4	flower bud of *P. quinquefolius*	Jilin	Baishan	20220703
PQF-5	flower bud of *P. quinquefolius*	Jilin	Baishan	20220901
PQF-6	flower bud of *P. quinquefolius*	Jilin	Baishan	20221008
PQF-7	flower bud of *P . quinquefolius*	Liaoning	Xinbin	20220801
PQF-8	flower bud of *P. quinquefolius*	Liaoning	Xinbin	20220907
PQF-9	flower bud of *P. quinquefolius*	Liaoning	Xinbin	20221003
PNF-1	flower bud of *P. notoginseng*	Yunnan	Wenshan	20220806
PNF-2	flower bud of *P. notoginseng*	Yunnan	Wenshan	20220902
PNF-3	flower bud of *P. notoginseng*	Yunnan	Wenshan	20221011
PNF-4	flower bud of *P. notoginseng*	Yunnan	Qiubei	20220701
PNF-5	flower bud of *P. notoginseng*	Yunnan	Qiubei	20220809
PNF-6	flower bud of *P. notoginseng*	Yunnan	Qiubei	20220911
PNF-7	flower bud of *P. notoginseng*	Yunnan	Yanshan	20220906
PNF-8	flower bud of *P. notoginseng*	Yunnan	Yanshan	20221010
PNF-9	flower bud of *P. notoginseng*	Yunnan	Yanshan	20221113

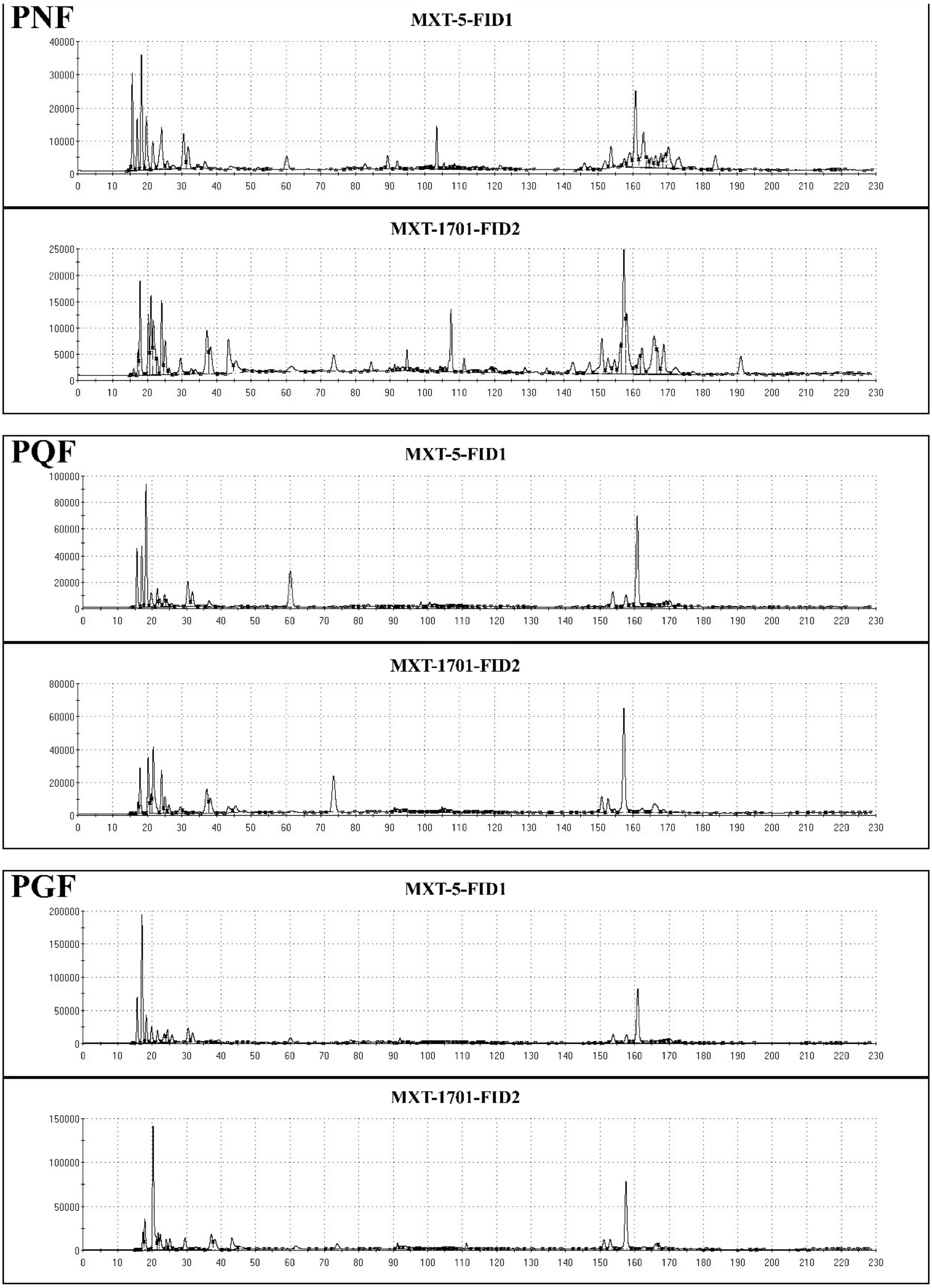

Figure S1. (A) The total ion chromatogram (TIC) of the flower bud of PGF, PQF and PNF aroma compounds identified by fast GC e-nose (MXT-5-FID1 and MXT- 1701-FID2).

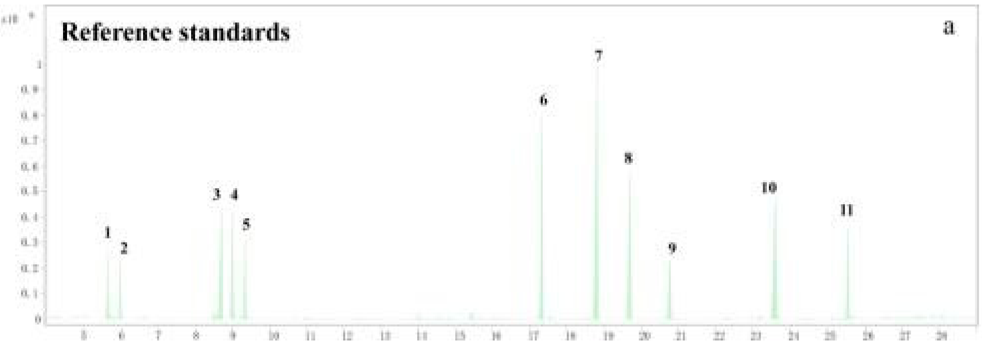

Figure S2. Reference compounds spectrogram.
